# Ovarian abscess caused by *Salmonella enterica* serovar Typhi: a case report

**DOI:** 10.1186/s13256-019-2229-y

**Published:** 2019-09-25

**Authors:** Aneley Getahun S., Josese Limaono, Raween Ligaitukana, Orisi Cabenatabua, Vika Soqo, Raape Diege, Mikaele Mua

**Affiliations:** 1 School of Public Health, College of Medicine Nursing and Health Science, Fiji National University, Suva, Fiji; 2 The Peter Doherty Institute for Infection and Immunity, University of Melbourne, Melbourne, VIC 3000 Australia; 3 Labasa Hospital, Ministry of Health and Medical Services, Labasa, Fiji; 4 Northern Health Services, Ministry of Health and Medical Services, Labasa, Fiji

**Keywords:** *Salmonella* Typhi, Typhoid fever, Ovarian abscess, Fiji

## Abstract

**Background:**

Typhoid fever is a human-specific disease caused by a bacterium, *Salmonella enterica* subspecies *enterica* serovar Typhi. It is transmitted through ingestion of contaminated food or water. It is mostly diagnosed by blood culture. *Salmonella* Typhi usually manifests as a febrile illness with bacteremia after initial entry through the gastrointestinal route, but it can occasionally cause significant disease in extraintestinal sites. We report a case of a girl in Fiji with a right ovarian abscess infected by *Salmonella* Typhi.

**Case presentation:**

A 14-year-old iTaukei (indigenous Fijian) girl presented to our hospital with abdominal pain of 1 month’s duration. Two days prior to her admission, she developed high-grade fever and nausea and had one episode of vomiting. On presentation, she appeared unwell; she was tachycardic (116 beats per minute) and febrile (38.8 °C). Her abdominal examination revealed generalized tenderness. Other examination findings were normal. The provisional diagnosis of abdominal sepsis led to an emergency laparotomy during which an enlarged right ovary was found to be spontaneously discharging pus. The ovary was incised and drained, and the patient was commenced on intravenous ceftriaxone 1 g twice daily, cloxacillin 1 g four times daily, and metronidazole 500 mg three times daily. She recovered promptly and was discharged to home on the sixth postoperative day. The purulent material from the ovary grew *Salmonella* Typhi.

**Conclusion:**

Extraintestinal infections caused by *Salmonella* Typhi are rare but can cause severe and life-threatening disease. Our patient had a prolonged history of abdominal pain and was found to have a ruptured right ovarian abscess due to *Salmonella* Typhi. Ovarian abscesses in girls who are not sexually active are not associated with pelvic inflammatory disease and suggest local or hematogenous spread. This case report may increase health workers awareness to include common and endemic infections in the differential diagnosis of unusual clinical presentations to help the initiation of appropriate investigation and management as quickly as possible.

## Background

*Salmonella enterica* subspecies *enterica* serovar Typhi is a human-specific bacterium that causes a systemic infection known as *typhoid fever*. Humans acquire *Salmonella* Typhi through ingestion of contaminated food or water. The common clinical manifestations of typhoid fever include fever; headache; and gastrointestinal symptoms such as abdominal pain, diarrhea, constipation, vomiting, and loss of appetite [1]. On rare occasions, *Salmonella* Typhi infection may spread to other organs such as the brain, bones, joints, and ovaries through hematogenous spread, or intestinal wall infection may directly spread and cause infection in surrounding structures [2, 3]. Infection of reproductive organs by *Salmonella* Typhi is rare even in endemic areas [2]. We report a case of a right ovarian abscess caused by *Salmonella* Typhi in a 14-year-old girl in Fiji.

## Case presentation

### History of present illness

A 14-year-old iTaukei (indigenous Fijian) girl presented to our hospital with a 1-month history of abdominal pain. Two days prior to her admission, her abdominal pain became worse, especially after movement, and was relieved by lying down. She also developed a high-grade fever and nausea and had one episode of vomiting. She gave no history of cough, diarrhea, vomiting, or dysuria. Her last menstrual period was on 28/9/2017. She denied any history of sexual intercourse.

She was referred to a nearby hospital, where ultrasonography showed a cystic structure measuring 4.9 cm × 4.4 cm at the right adnexal region. Minimal free fluid was seen in the pouch of Douglas. No obvious appendix abnormality was seen, and other structures, including the uterus, were normal. She was then transferred to one of the main hospitals in Fiji for further investigation and management.

### Past medical and social history

The patient’s past medical history was unremarkable. She had no record of previous admission. She had not been receiving any regular medication and had no known allergies.

The patient resides in a village that is approximately a 45-minute drive from the nearest town. She lives in a two-bedroom corrugated house with her parents and three younger siblings. Her usual diet consists of boiled root crops (such as cassava and taro), local vegetables, and fish. The water source is a spring that supplies the whole village. The family uses a water seal toilet. She gave no history of travel outside her village in 2017.

### Physical examination

On examination at the main hospital, the patient looked unwell. Her pulse was 116 beats per minute, blood pressure 114/60 mmHg, respiratory rate 21 breaths per minute, and temperature 38.8 °C. The results of her chest and cardiovascular examinations were normal. Abdominal examination revealed generalized tenderness on light palpation. However, there was no guarding or rebound tenderness, and no mass was palpable. The result of the per rectal examination was normal, as was the remaining examination.

Blood tests revealed a hemoglobin of 9.8 g/dl (normal range [NR] 11.5–18.5 g/dl) and white blood cell count of 14,100 cells/mm^3^ (NR 4000–11,000 cells/mm^3^). Two blood cultures revealed no growth after 48 hours. The patient’s full blood count, liver and renal function, and serum electrolyte test results are shown in Table 1. The results of her chest and abdominal x-ray were normal.

**Table 1:** Results of laboratory investigations

	28/10/2017 (admission)	29/10/2017	30/10/2017	Labasa Hospital laboratory reference
Hemoglobin	9.8	8.3	11.1	11.5–18.5 g/dl
WBC	14,100	10,950	10,060	4000–11,000 cells/mm^3^
Platelet count	313,000	289,000	329,000	140,000–450,000 cells/mm^3^
Urea	3.0	3.2	2.6	2.8–7.2 mmol/L
Creatinine	56	34	35	44.0–106.0 μmol/L
Total bilirubin	22	8	7	2–21 μmol/L
ALP	315	196	189	30–120 U/L
AST	41	18	19	0–31 U/L
ALT	42	22	18	0–34 U/L
Sodium	139	135.9	135.4	135.0–148.0 mmol/L
Potassium	3.9	3.8	3.9	3.50–5.30 mmol/L
Chloride	102	104.3	105.5	90.0–110.0 mmol/L

*WBC* white blood cell count, *ALP* alkaline phosphatase, *AST* aspartate aminotransferase, *ALT* alanine aminotransferase

**Fig. 1:**
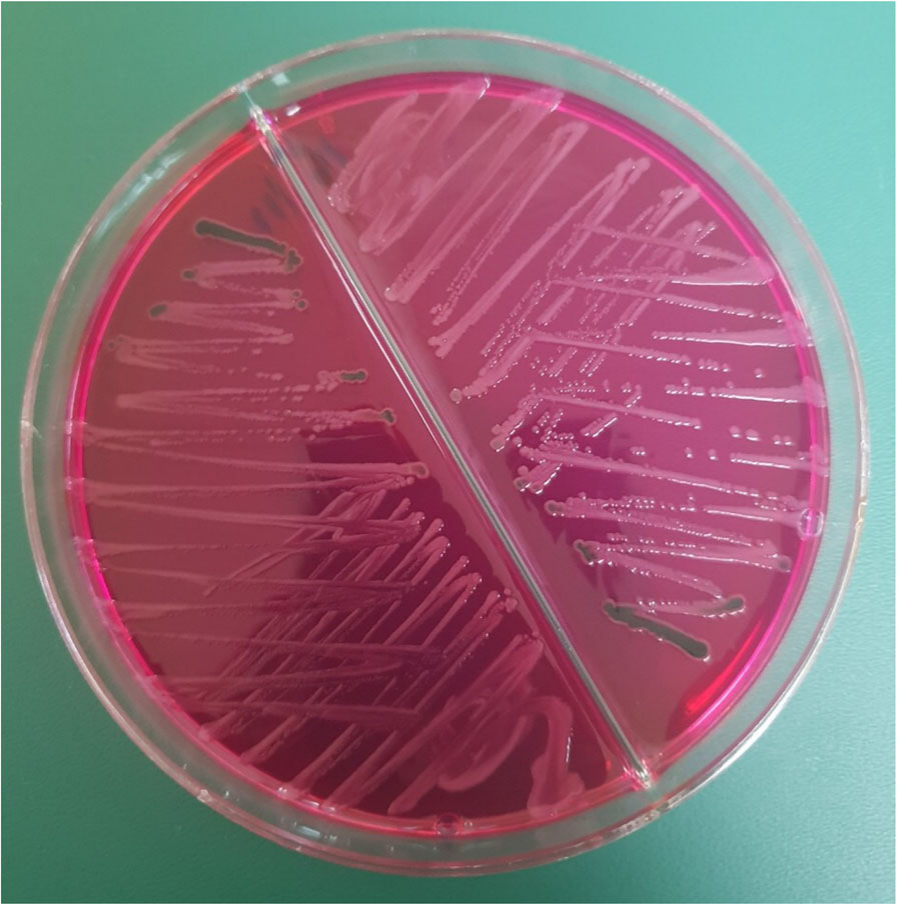
*Salmonella* Typhi colonies on Xylose Lysine Deoxycholate (XLD) agar

Exploratory laparotomy revealed serosal appendicitis with erythema and abundant fibrinous peritoneal fluid; hence, an appendicectomy was performed. It was noted that the right ovary was enlarged and had ruptured because of pus collection. The right ovary was incised, and pus was drained. This pus was cultured and yielded a pure growth of S*almonella* Typhi (Fig. 1), which was identified by using Microbact™ 12A/12B identification kits (Oxoid Microbiology Products, Altrincham, UK). This identification was later confirmed by *Salmonella*-specific antiserum testing (Difco™; Becton, Dickinson and Company, Franklin Lakes, NJ, USA). The antimicrobial sensitivity test was performed using a disk diffusion method on Mueller-Hinton agar. The organism was susceptible to all tested antibiotics (ampicillin, chloramphenicol, trimethoprim-sulfamethoxazole, gentamicin, cephalothin, ceftriaxone, ciprofloxacin, and nalidixic acid). Histopathology of the resected appendix revealed reactive lymphoid follicle in mucosa and acute inflammation on the serosal layer, compatible with periappendicitis.

### Treatment and follow-up

The patient was treated with intravenous ceftriaxone 1 g twice daily, cloxacillin 1 g four times daily, and metronidazole 500 mg three times daily for 5 days. She made an uneventful recovery and was discharged to home on the sixth postoperative day to complete a further 8 days of oral cotrimoxazole. Patient was reviewed 1 week after her discharge from the hospital. She did not have any complaint; her surgical wound was clean; and there were no remarkable physical findings. The result of her stool culture after completion of treatment was negative for *Salmonella*.

## Discussion and conclusion

In this report, we describe a case of right ovarian abscess caused by *Salmonella* Typhi in a 14-year-old girl with no history of sexual intercourse. Tubo-ovarian abscess is one of the late and severe complications of pelvic inflammatory disease (PID) that is commonly caused by ascending infection from the lower genital tract, such as the cervix or vagina [4]. Tubo-ovarian abscess also can occur without a previous episode of PID or sexual activity through local spread from an infected adjacent organ such as the appendix or the small intestine or by hematogenous spread following bacteremia [5–7].

Typhoid fever is endemic in Fiji [8–10]. To our knowledge, this is the first reported case of ovarian abscess caused by *Salmonella* Typhi in Fiji. Our patient came from a known typhoid-endemic area where nine laboratory-confirmed cases were reported between October and December 2017. The most probable route of infection in this patient is hematogenous spread after a recent typhoid exposure with a subclinical bacteremia phase. Local spread from infected bowel is less likely in the absence of significant gastrointestinal symptoms and lack of small bowel inflammation in laparotomy.

Extraintestinal manifestations of *Salmonella* Typhi are not common even in endemic areas. A few cases of ovarian abscess caused by *Salmonella* Typhi have been reported from India [11, 12], Nepal [13], Malta [14], and Spain [15]. Risk factors for ovarian infections are not fully understood. A review of *Salmonella* infection of the reproductive organ and case reports showed most of the cases to have underlying structural abnormalities such as ovarian or dermoid cysts or to be in patients who were immunocompromised [2, 11, 12]. Generally, *Salmonella* infection involving the reproductive tract has a good prognosis [3]. However, complications such as ruptured abscess with peritonitis have been reported from India and resulted in death [11].

The occurrence of ovarian abscess in a young, sexually nonactive girl poses a significant diagnostic dilemma, especially in a primary care setting with limited investigation capacity. As seen in our patient, the clinical features of ovarian abscess complicated by *Salmonella* Typhi are nonspecific and can mimic an acute abdomen presentation such as appendicitis or PID. Sudden onset of severe pain with fever and vomiting after a 4-week history of abdominal pain signifies complicated disease (for example, a perforated viscus or abscess formation); hence, our patient was urgently transferred to a regional center for surgery.

The diagnosis of ovarian abscess involves thorough history taking and physical examination to rule out the various differential diagnoses. Laboratory investigations are also useful in the diagnosis. Leukocytosis is common; however, it is not specific. In only one systematic review, half of the patients with salmonella genital infection (including the ovaries) had an elevated white blood cell count [3]. Pelvic ultrasound is an important tool for detection of any enlargement of the ovaries and/or underlying anatomical abnormality, which can be a risk factor for abscess formation. Laparoscopic evaluation of the pelvic organs is considered the gold standard for diagnosing tubo-ovarian abscess [2, 4] . Definitive diagnosis should be made by isolating *Salmonella* Typhi from purulent exudates.

Treatment of ovarian abscess caused by *Salmonella* Typhi includes antibiotics as well as surgical drainage [3, 13]. The choice of antibiotics is generally guided by the susceptibility pattern of the local isolates of *Salmonella* Typhi [2]. The duration of treatment depends on the type of organ involved and the extent of complications. Cohen *et al.* [3] suggested 6 weeks of antibiotic treatment for male reproductive organ involvement. Huang and DuPont [2] proposed 7–14 days of treatment for genitourinary infection or longer if underlying complications such as stone or abscess collection occur. In our patient, *Salmonella* Typhi isolates were sensitive to all tested antibiotics. Our patient was treated with 5 days of intravenous ceftriaxone followed by oral cotrimoxazole for 8 days.

Although the majority of patients with typhoid fever present with systemic symptoms such as fever, headache, and gastrointestinal symptoms, health workers in Fiji should consider extraintestinal salmonella infection in their differential diagnosis of patients with deep-seated abscesses such as in the ovary. Ultrasonography, which is available in subdivisional hospitals in Fiji, could be used as an initial investigation of ovarian abscess. Isolation of *Salmonella* Typhi from purulent exudates confirms the diagnosis.

## Data Availability

Not applicable
